# Supplementary material for: Pan-Cancer Analysis of the Genomic Alterations and Mutations of the Matrisome
Source: Cancers (Basel). 2020 Jul 24;12(8):2046. doi: 10.3390/cancers12082046 (PMC7463652; doi:10.3390/cancers12082046)
Supplement: Supplementary file 1 [file cancers-12-02046-s001.pdf]

Supplementary Materials:

Pan-Cancer Analysis of the Genomic Alterations and Mutations of the Matrisome

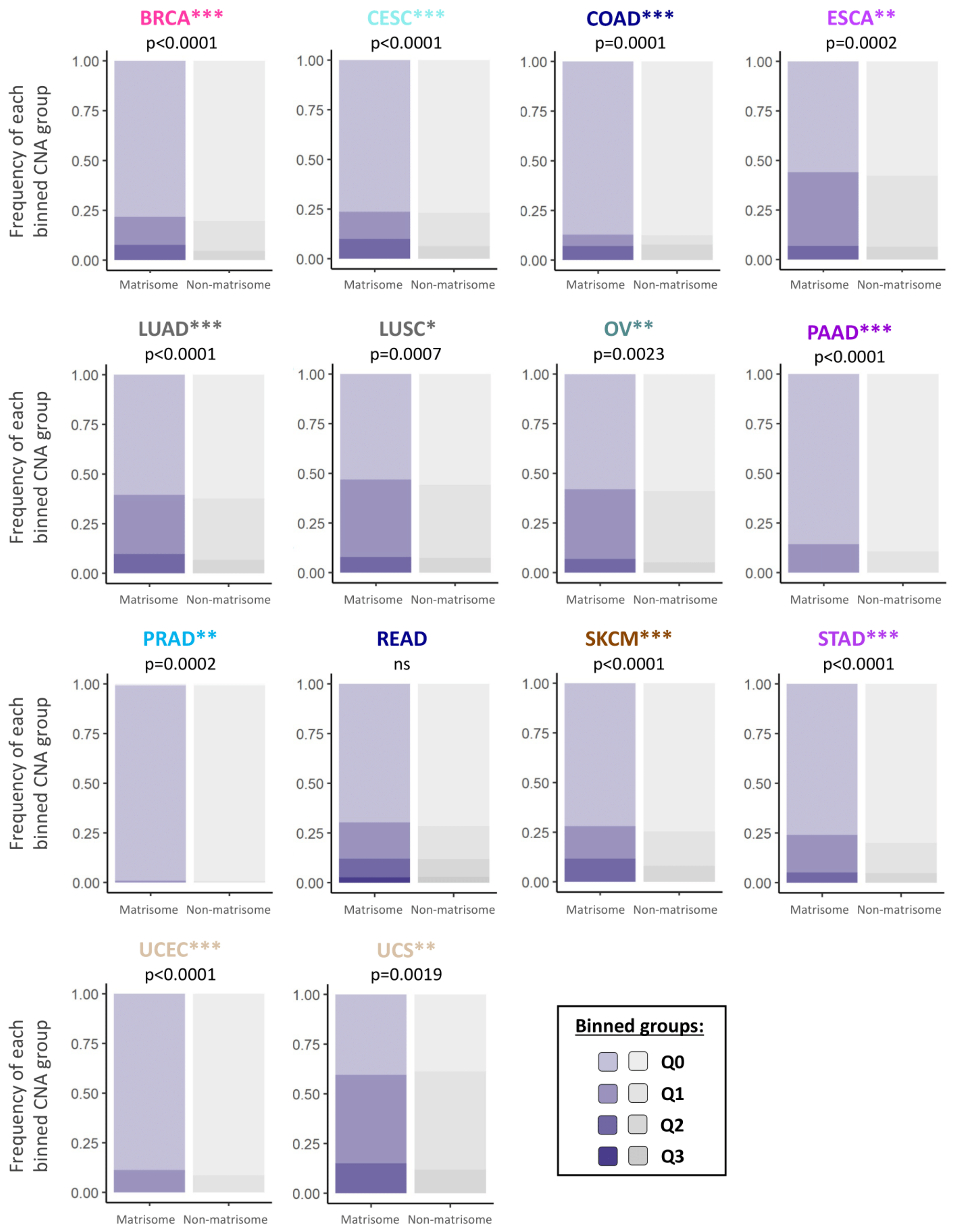

Figure S1A. Copy number alterations of matrisome genes across 14 different cancer types broken down by CNA type (related to Figure 1). Bar charts represent the frequency of shallow amplifications in

matrisome genes (purple bars) and non-matrisome genes (rest of the genome, grey bars) across 14 different cancer types. Chi-square test p-values are indicated for each CNA and cancer type (*$p < 0.05$; **$p < 0.01$; ***$p < 0.001$). Binned groups are represented by different shades of purple and grey to represents genes in which CNAs are found in x% of the samples: Q0 = 0% (lighter shade), 0% < Q1 ≤ 25%, 25% < Q2 ≤ 50%; 50% < Q3 ≤ 75%; Q4 > 75% (darker shade).

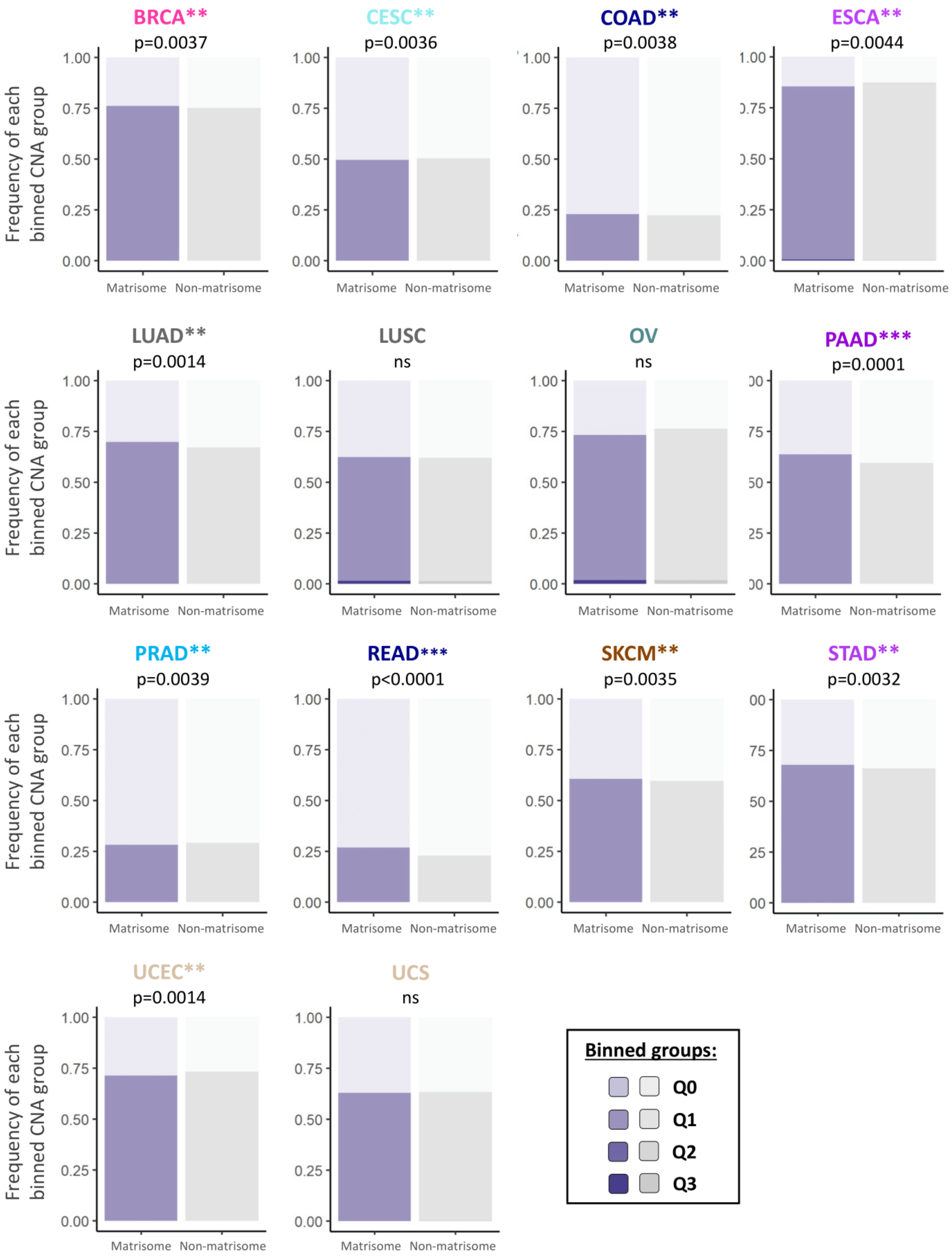

Figure S1B. Copy number alterations of matrisome genes across 14 different cancer types broken down by CNA type (related to Figure 1). Bar charts represent the frequency of deep amplification in matrisome genes (purple bars) and non-matrisome genes (rest of the genome, grey bars) across 14 different cancer types. Chi-square test p-values are indicated for each CNA and cancer type (*$p < 0.05$; **$p < 0.01$; ***$p <$

0.001). Binned groups are represented by different shades of purple and grey to represents genes in which CNAs are found in x% of the samples: Q0 = 0% (lighter shade), 0% < Q1 ≤ 25%, 25% < Q2 ≤ 50%; 50% < Q3 ≤ 75%; Q4 > 75% (darker shade).

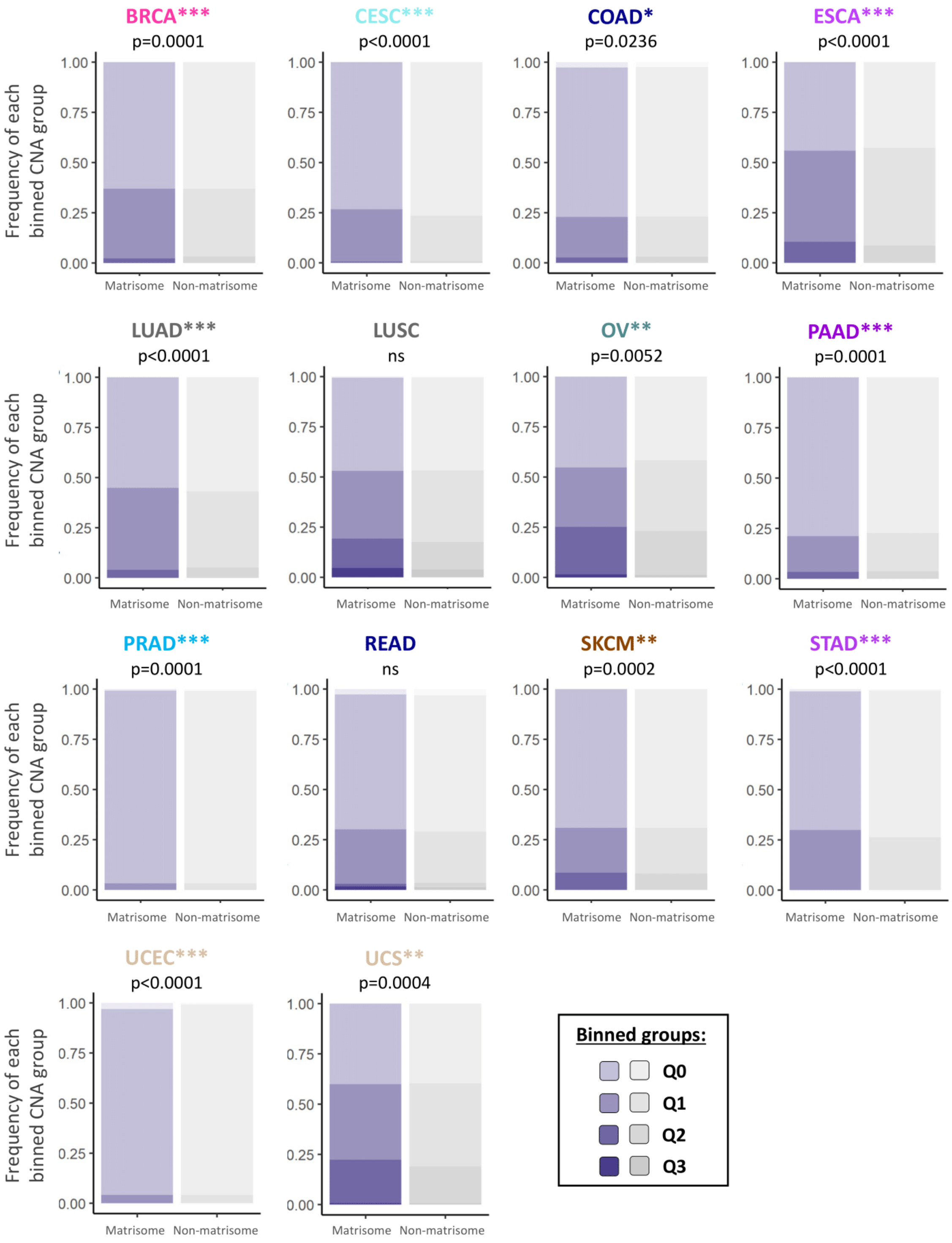

Figure S1C. Copy number alterations of matrisome genes across 14 different cancer types broken down by CNA type (related to Figure 1). Bar charts represent the frequency of shallow deletions in matrisome genes (purple bars) and non-matrisome genes (rest of the genome, grey bars) across 14 different cancer types. Chi-square test p-values are indicated for each CNA and cancer type (*$p < 0.05$; **$p < 0.01$; ***$p <$ 0.001). Binned groups are represented by different shades of purple and grey to represents genes in

which CNAs are found in x% of the samples: Q0 = 0% (lighter shade), 0% < Q1 ≤ 25%, 25% < Q2 ≤ 50%; 50% < Q3 ≤ 75%; Q4 > 75% (darker shade).

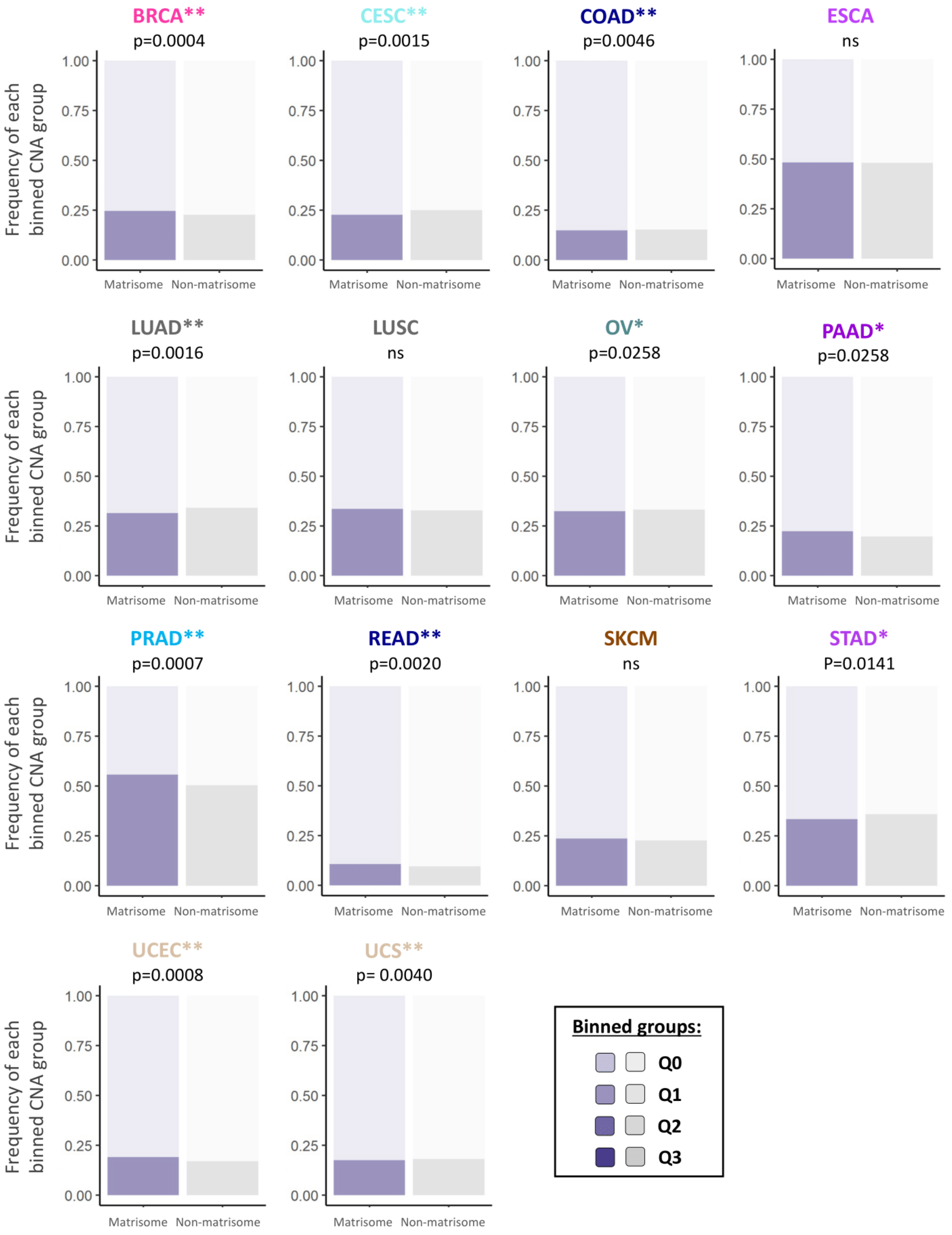

Figure S1D. Copy number alterations of matrisome genes across 14 different cancer types broken down by CNA type (related to Figure 1). Bar charts represent the frequency of deep deletions in matrisome genes (purple bars) and non-matrisome genes (rest of the genome, grey bars) across 14 different cancer types. Chi-square test *p*-values are indicated for each CNA and cancer type (**p* < 0.05; ***p* < 0.01; ****p* < 0.001). Binned groups are represented by different shades of purple and grey to represents genes in

which CNAs are found in x% of the samples: Q0 = 0% (lighter shade), $0\% < Q1 \leq 25\%$, $25\% < Q2 \leq 50\%$; $50\% < Q3 \leq 75\%$; $Q4 > 75\%$ (darker shade).

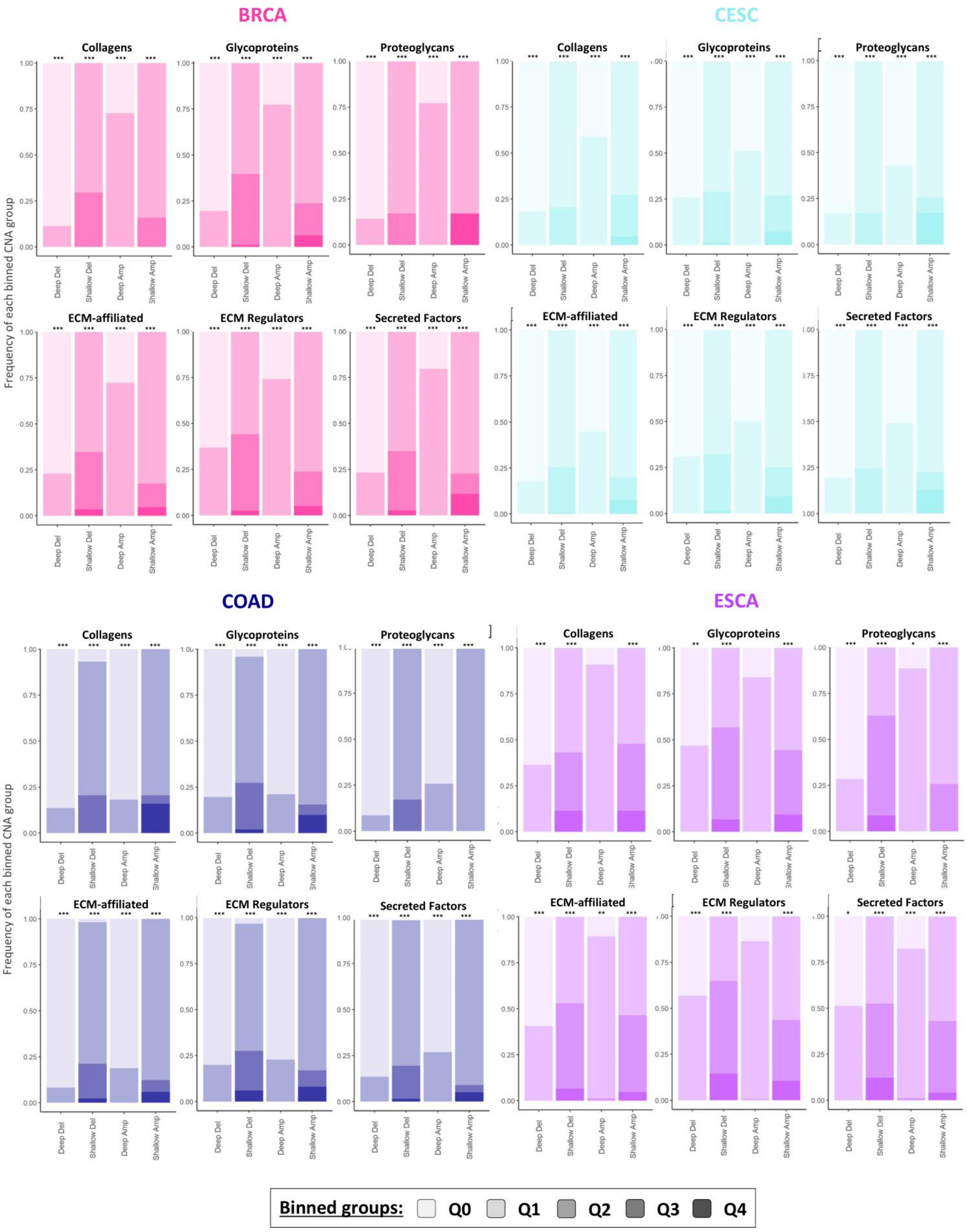

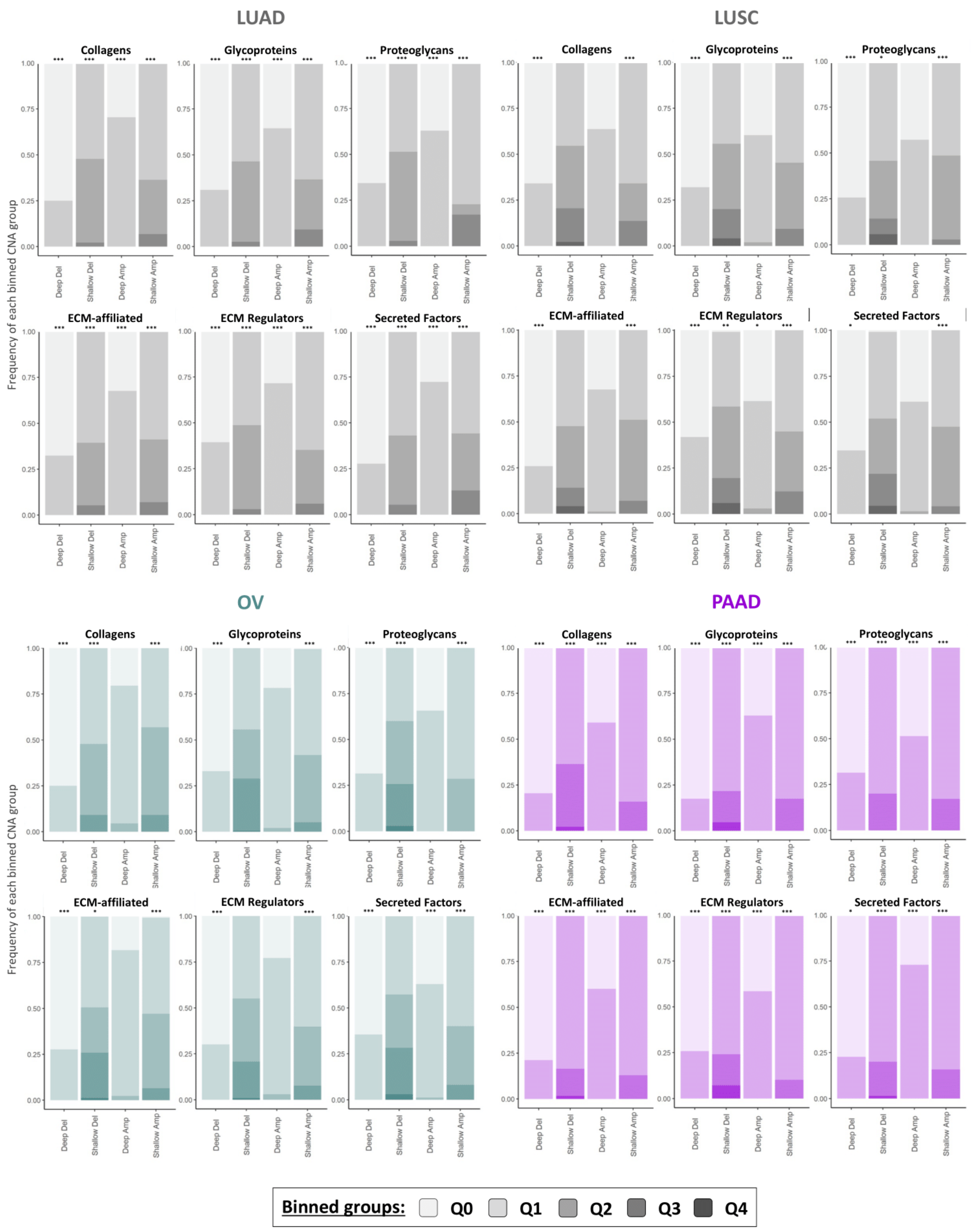

LUAD
LUSC
OV
PAAD
Collagens
Glycoproteins
Proteoglycans
ECM-affiliated
ECM Regulators
Secreted Factors
Frequency of each binned CNA group
Deep Del
Shallow Del
Deep Amp
Shallow Amp
Binned groups: Q0 Q1 Q2 Q3 Q4

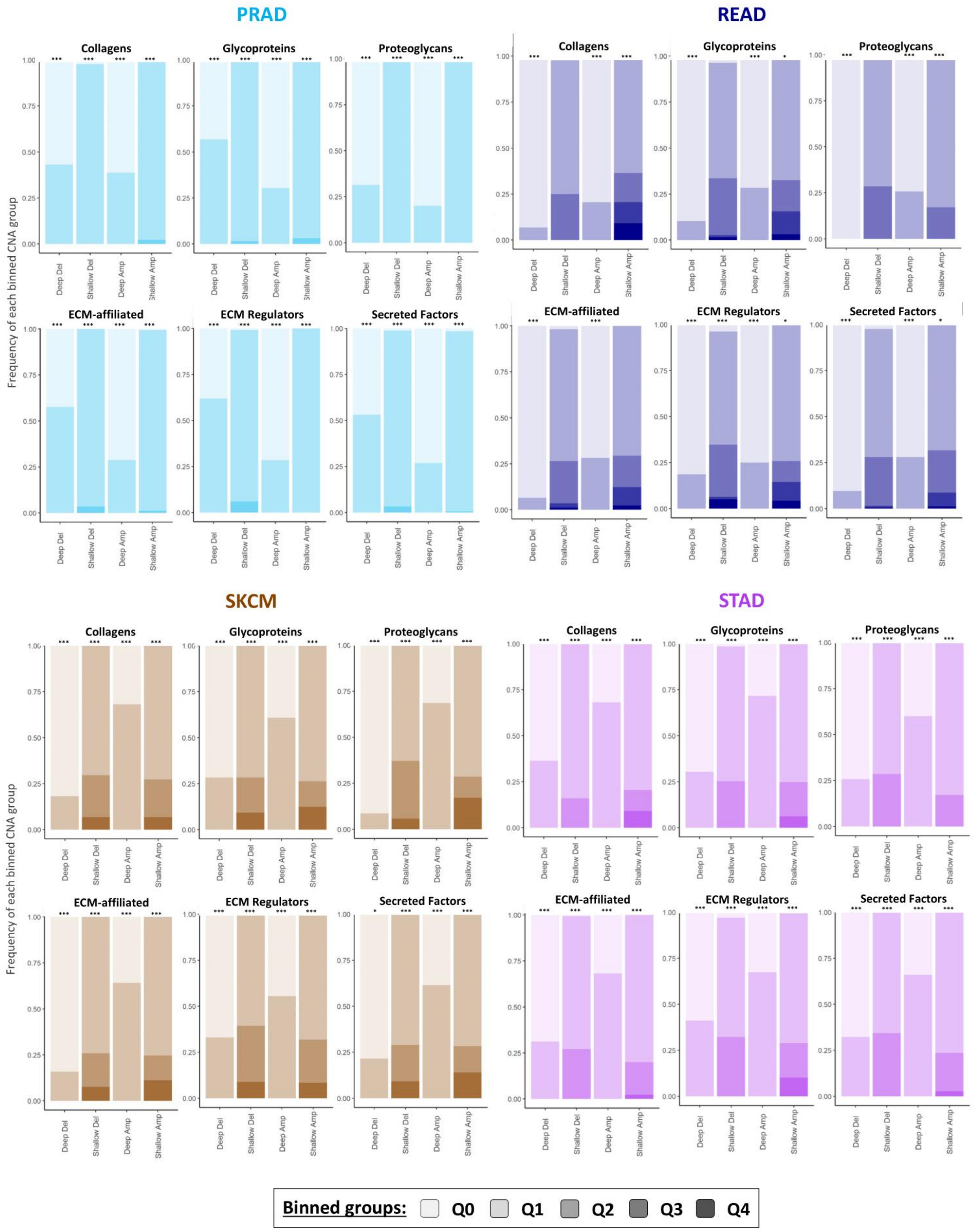
PRAD
READ
SKCM
STAD
Collagens
Glycoproteins
Proteoglycans
ECM-affiliated
ECM Regulators
Secreted Factors
Frequency of each binned CNA group
Deep Del
Shallow Del
Deep Amp
Shallow Amp
Binned groups: Q0 Q1 Q2 Q3 Q4

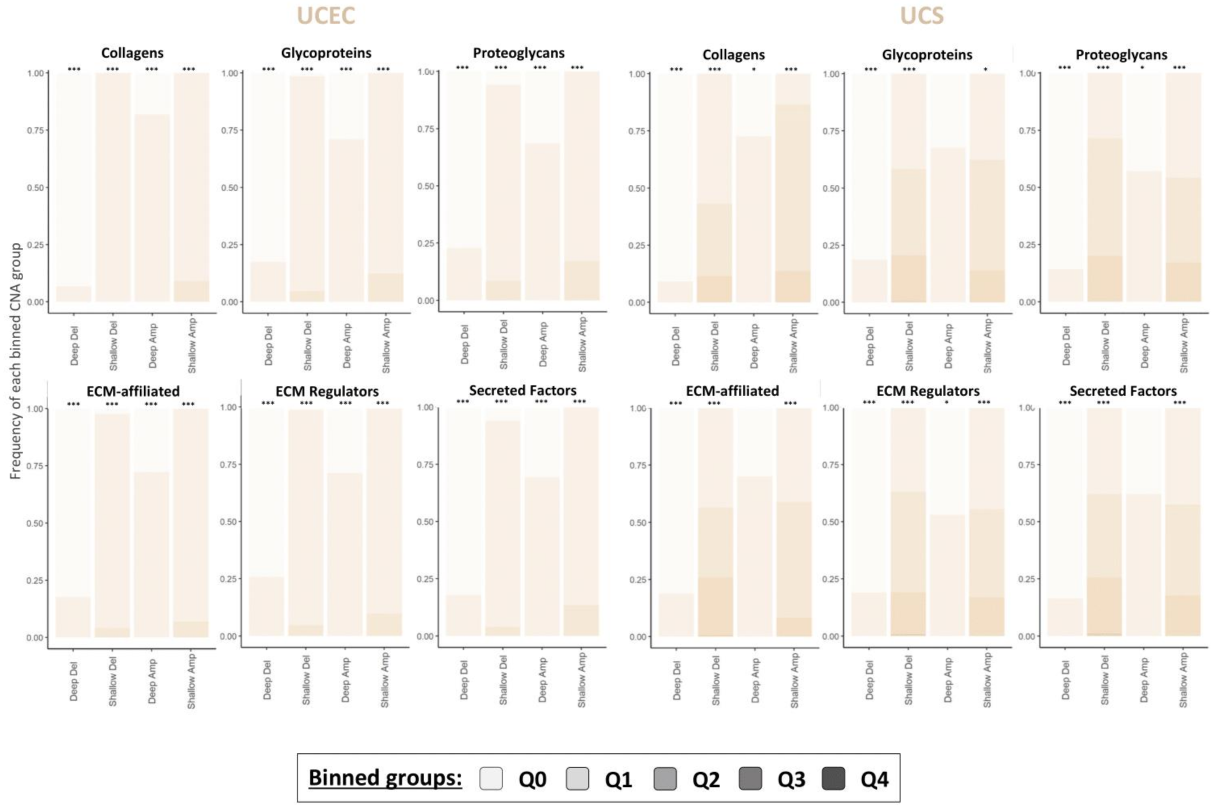

Figure S2. Copy number alterations of matrisome genes across 14 different cancer types broken down by CNA type and matrisome gene category (related to Figure 1). Bar charts represent the frequency of shallow amplifications, deep amplification, shallow deletions, and deep deletions in genes belonging to different matrisome categories across 14 different cancer types. Chi-square test *p*-values are indicated for each matrisome gene category, each CNA type, and each cancer type (*$p < 0.05$; **$p < 0.01$; ***$p < 0.001$). Binned groups are represented by different shades to represents genes in which CNAs are found in x% of the samples: Q0 = 0% (lighter shade), 0% < Q1 ≤ 25%, 25% < Q2 ≤ 50%; 50% < Q3 ≤ 75%; Q4 > 75% (darker shade).

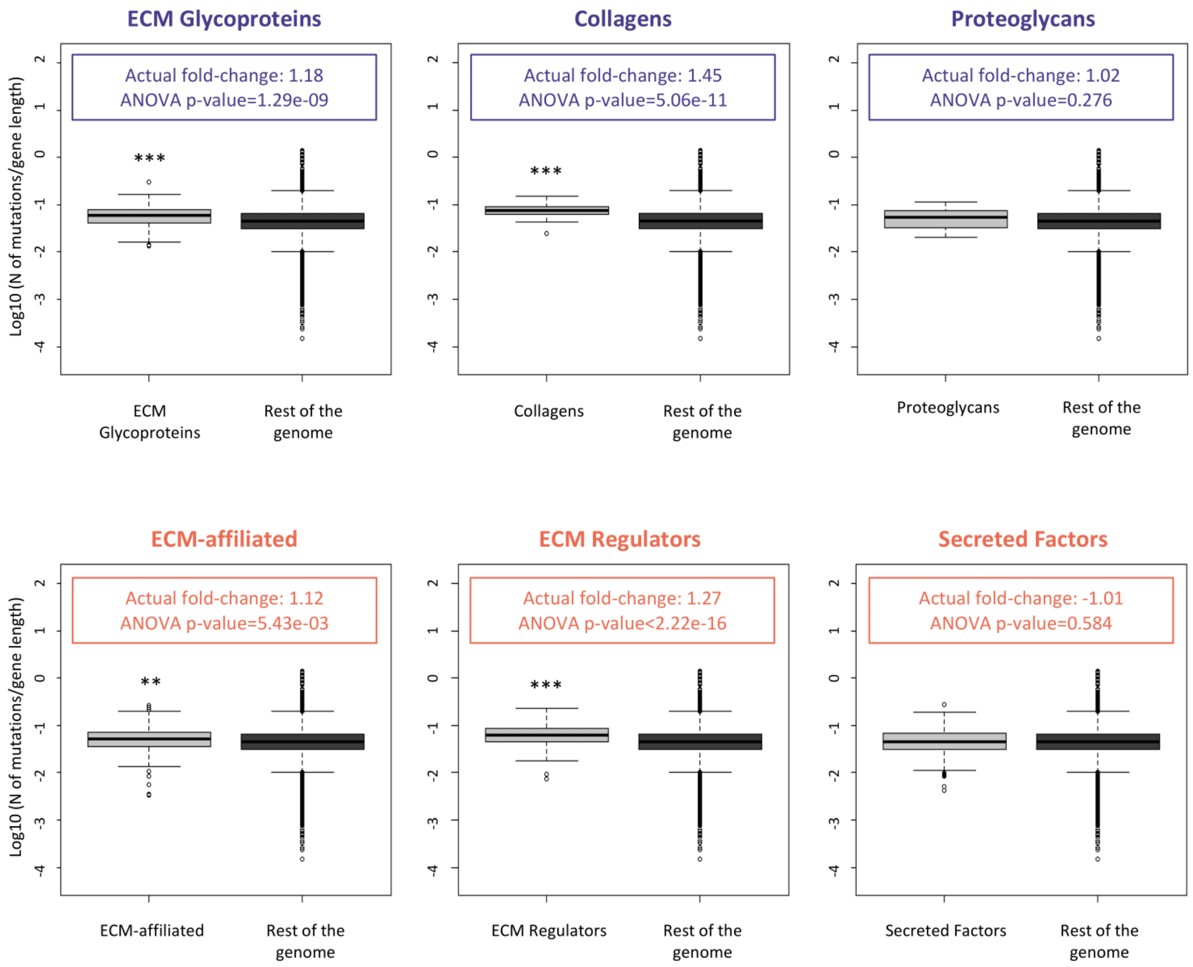

Figure S3. Number of mutations per matrisome gene length and matrisome gene category (related to Figure 3). Box plots represent the number of genes of given number of mutations per gene length ratios for core matrisome (upper panels) and matrisome-associated genes (lower panels). ** $p < 0.01$; *** $p < 0.001$)

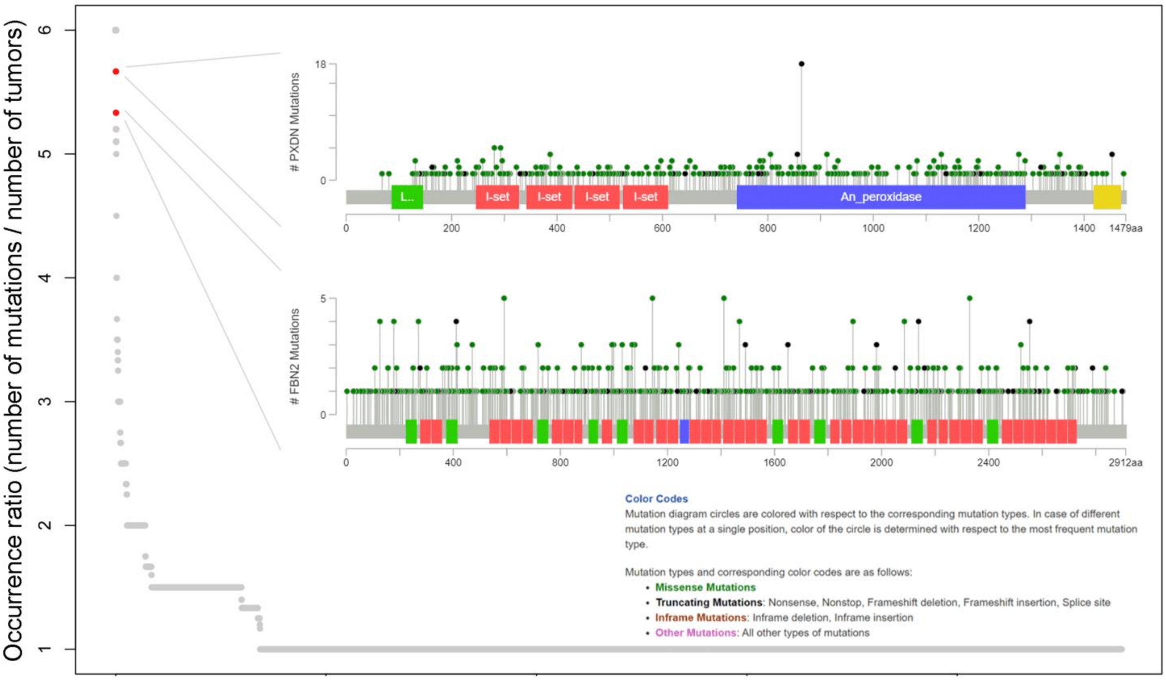

Figure S4. Identification of potential mutational hot spots in matrisome genes. Potential hotspots were defined as those mutations occurring in at least three patients in at least two different tumor types, and their abundance is reported as the ratio of total occurrences in the PanCancer cohort divided by the number of tumors in which the mutation occurs. Genes marked in red (candidate hotspot mutations) have total occurrences between 10 and 17 and appear in 2 or 3 tumor types. Missense mutations (green circles) and truncation mutations, including nonsense mutations, nonstop mutations, frameshift deletions, frameshift insertions or splice sites (black circles) are shown for each gene. Data and graphs for the lollipop graphs are from cBioPortal.

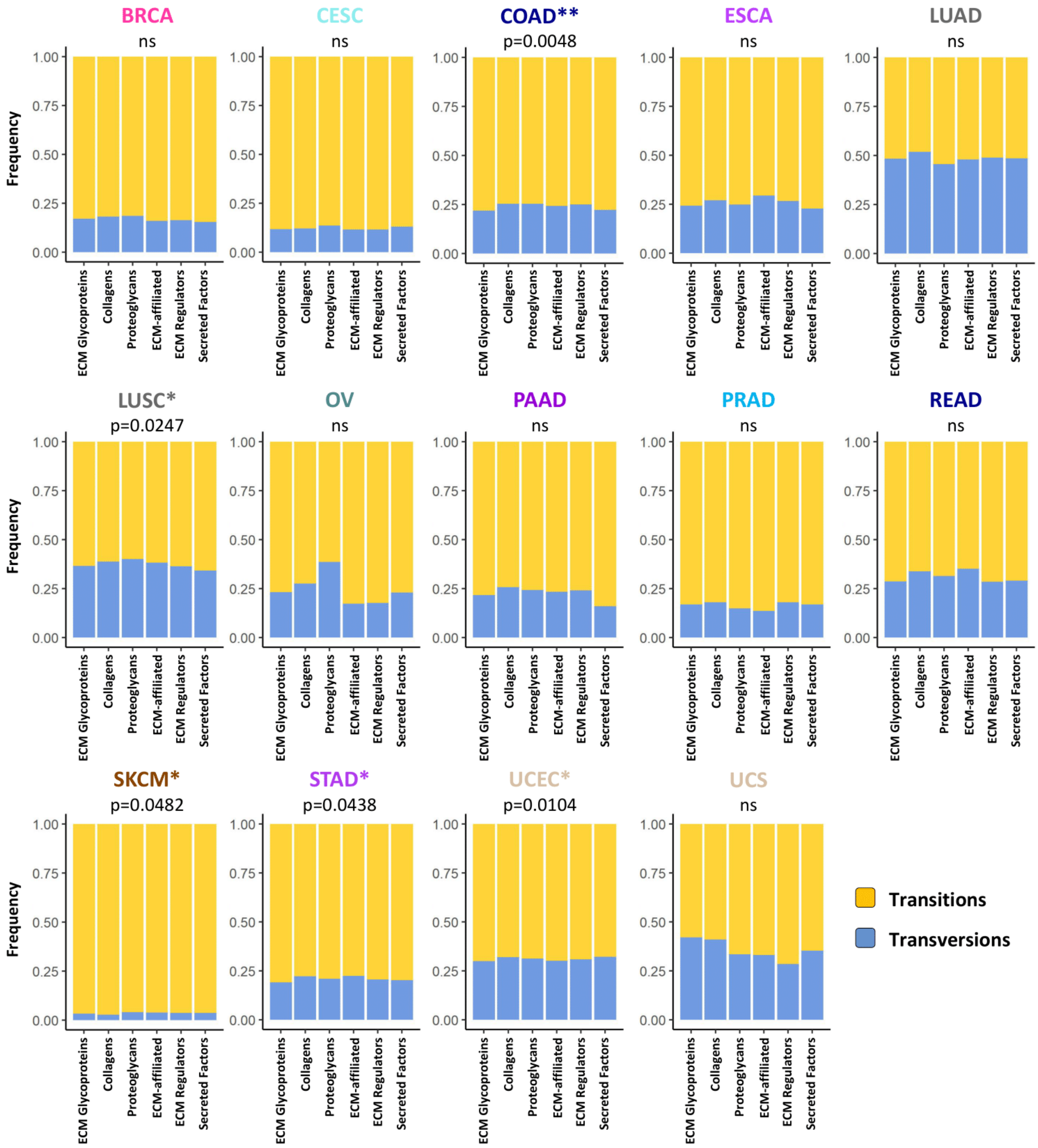

Figure S5. Type of mutations per matrisome gene category and cancer type. Bar charts represent the frequency of transversions (blue) or transition (yellow) affecting matrisome genes and their frequency across cancer types. (*$p < 0.05$; **$p < 0.01$)

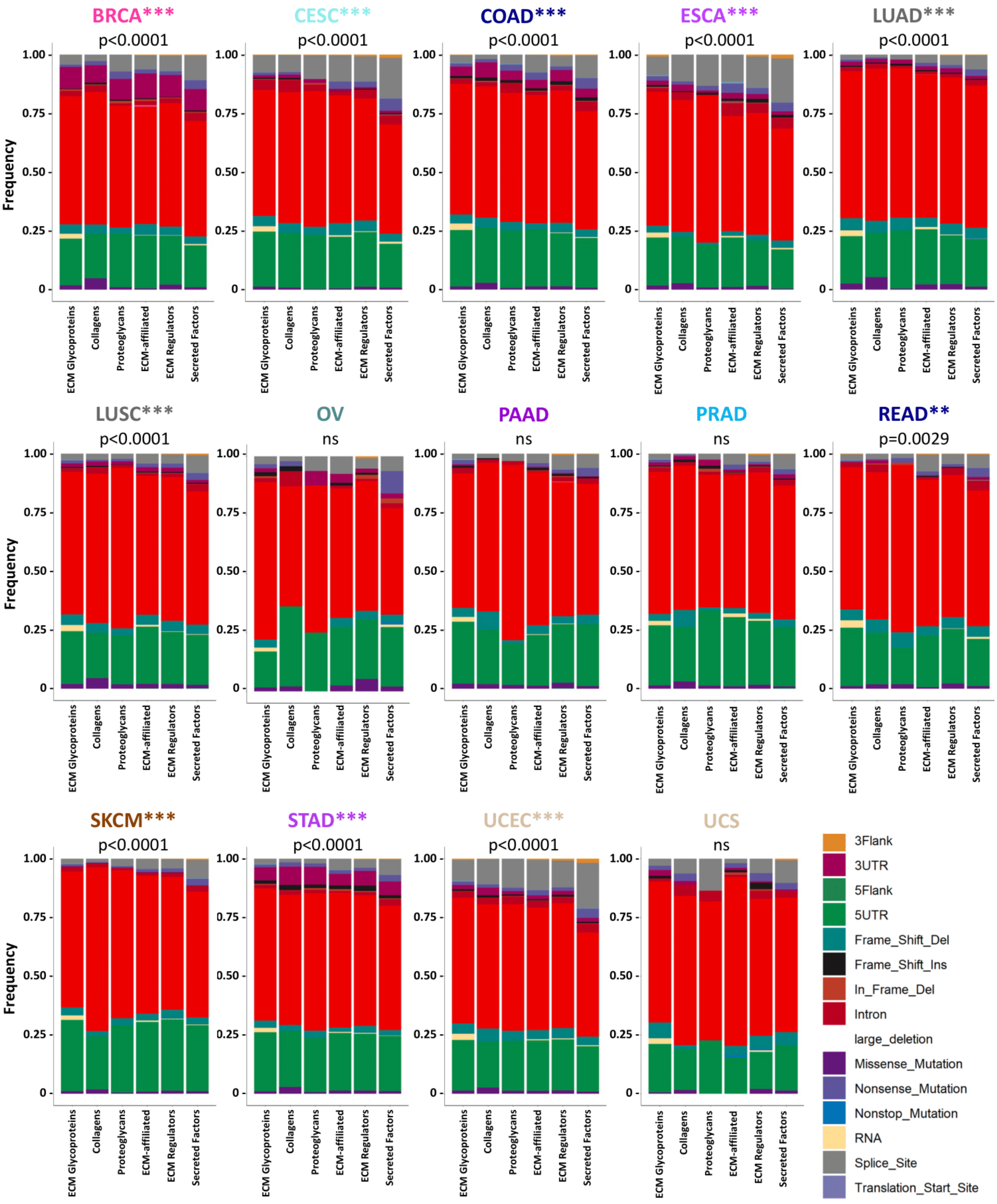

Figure S6. Location and type of mutations per matrisome gene category and cancer type. Bar charts represent the site and type of mutation affecting matrisome genes and their frequency across cancer types. $^{**}p < 0.01$; $^{***}p < 0.001$

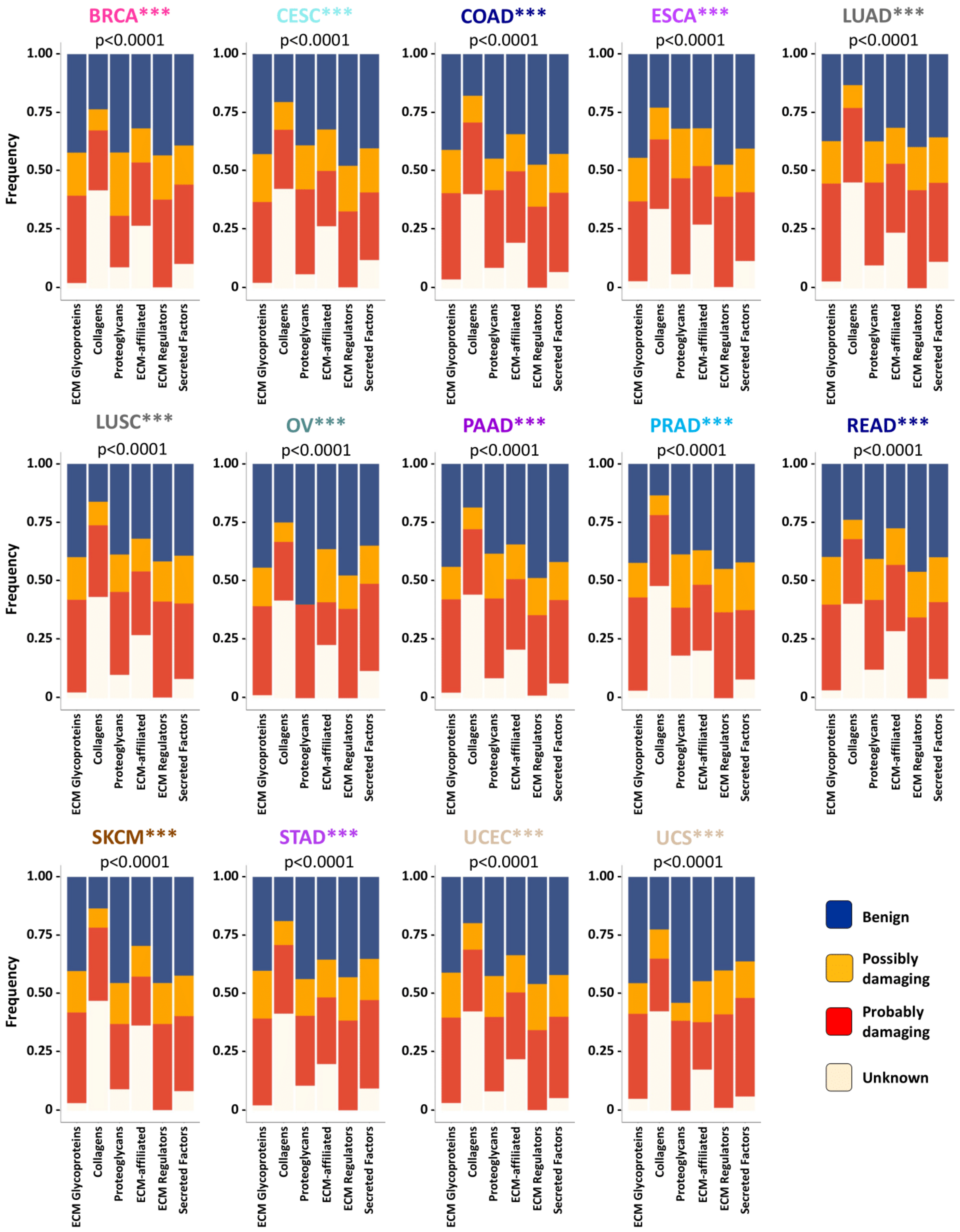

Figure S7. Prediction of mutational effects per matrisome gene category and cancer type (related to Figure 5). Bar charts represent the frequency of predicted mutational effect of matrisome genes on function, per matrisome gene category and cancer type. Color code is as follows: benign (blue), possible damaging (yellow), probably damaging (red), and unknown (light pink). ***$p < 0.001$

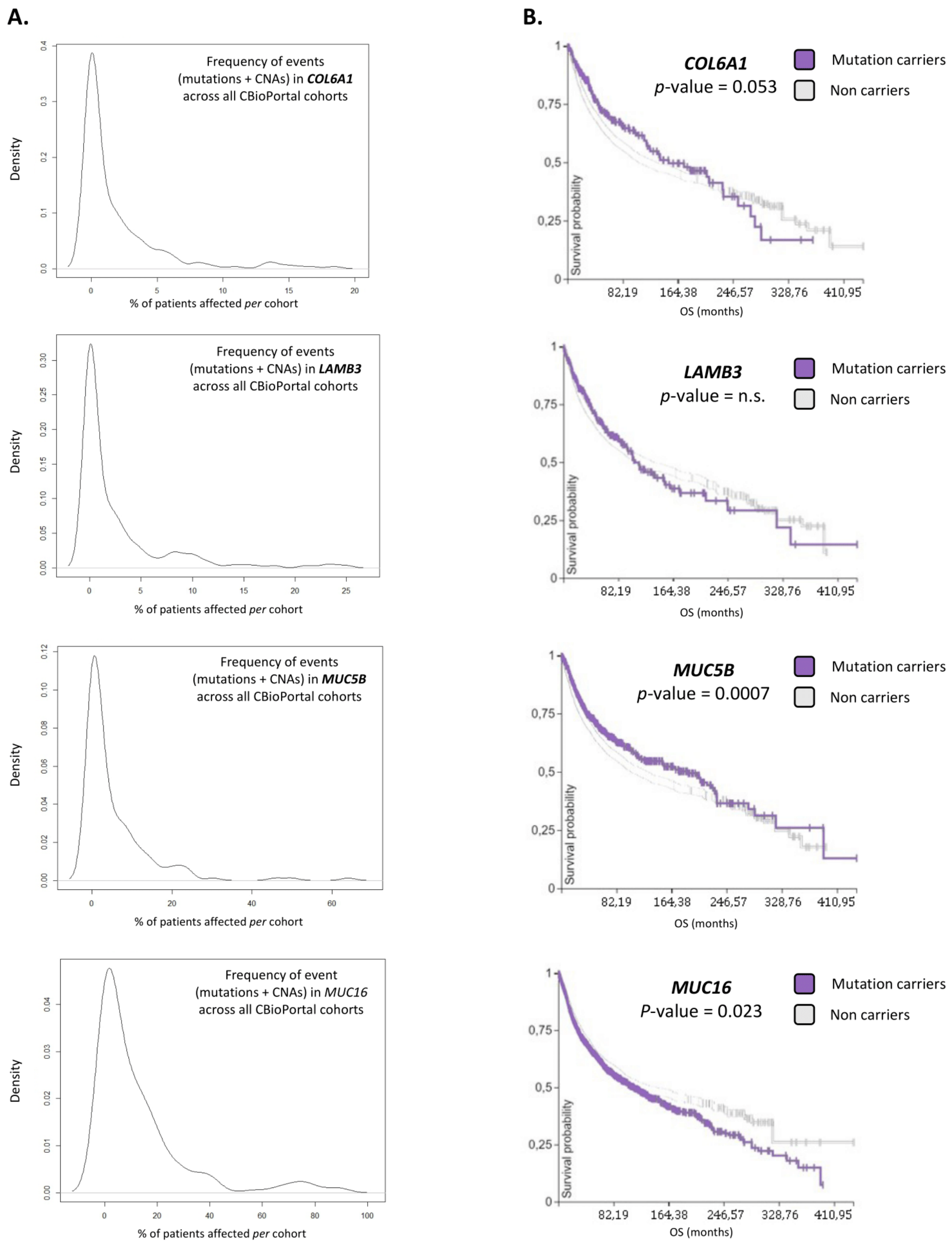

Figure S8. Cross-validation using independent cancer patient cohorts. (**A**.) Density plots represent the frequency of mutations and CNAs for the specified genes, *COL6A1*, *LAMB3*, *MUC5B*, or *MUC16* across all cohorts retrieved from the cBioPortal. (**B.**) Kaplan-Meier curves represent the overall survival probability over time (in months) of patients carrying (purple trace) or not (gray trace) mutations in the specified core matrisome genes *COL6A1* and *LAMB3*, or matrisome-associated genes *MUC5B* or *MUC16*. *p*-Values indicated correspond to the ones calculated in univariate analyses.

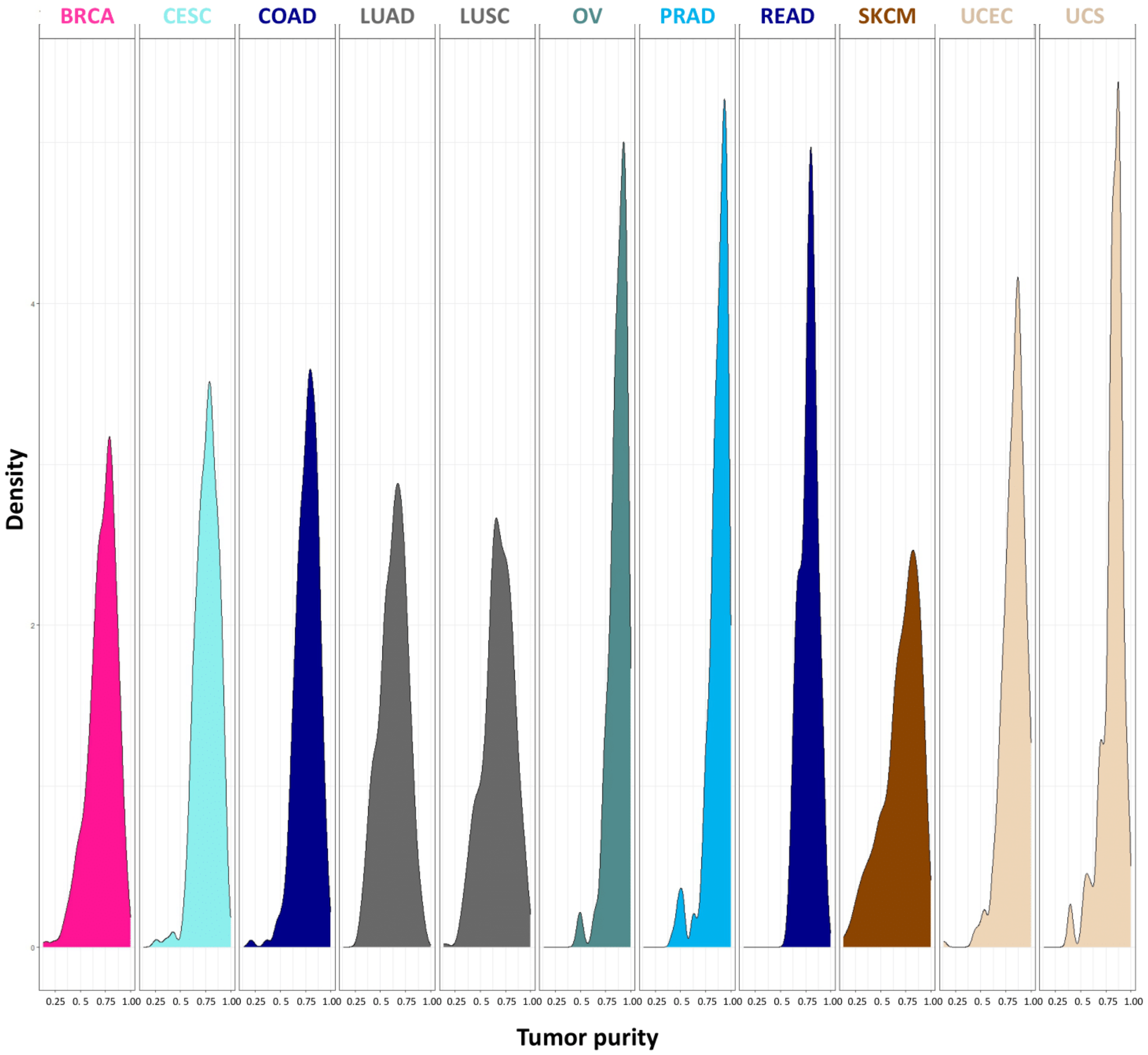

Figure S9. Purity of samples assessed across the TCGA Pan-Cancer cohort. Tumor purity across 11 of the 14 tumor types presented in this study, expressed as CPE (consensus measurement of purity estimations; see Methods for details). Density plots report the distribution of CPE measurement across each tumor type. At the time of the study, CPE data were not available for esophageal carcinoma (ESCA), stomach carcinoma (STAD) or Pancreatic Adenocarcinoma (PAAD).

Table S1. Consequences of CNAs on matrisome gene expression levels.

Table S2. Frequency and recurrence of mutations of matrisome genes

Table S3. Top 20 most frequently mutated domains in ECM proteins.

Table S4. Top 10 most mutated matrisome genes.

Table S5. Effect of mutations on univariate and multivariate survival at the ECM gene level (**A**) and ECM protein-domain level (**B**).

Table S6. Frequency of CNAs and mutations in the matrisome genes *COL6A1* (**A**), *LAMB3* (**B**), *MUC5B* (**C**), *MUC16* (**D**) in 180 different patient cohorts, representing 47500 cases available via cBioPortal.

Table S7. Correlation of tumor purity with occurrence of CNAs and mutations in matrisome genes.
